# Report of cold agglutinins in a patient with acute ischemic stroke

**DOI:** 10.1186/s12883-015-0482-2

**Published:** 2015-10-30

**Authors:** Haiqiang Jin, Wei Sun, Yongan Sun, Yining Huang, Yunchuang Sun

**Affiliations:** Department of Neurology, Peking University First Hospital, No.8 Xishiku Street, Xicheng District, Beijing 100034 PR China

**Keywords:** Cold agglutinin disease, Acute ischemic stroke

## Abstract

**Background:**

Studies on the role of cold agglutinins in the pathogenesis of acute ischemic stroke are scarce. We present a case of an elderly man with acute cerebral infarction probably due to cold agglutinin disease.

**Case presentation:**

On a cold morning, a 71-year-old male of Han nationality with a complaint of sudden onset left-sided weakness and difficulty in speaking was brought to the emergency department. Diffusion weighted magnetic resonance imaging of the brain showed a high-intensity area in the right basal ganglia and corona radiata. Laboratory test showed the presence of high titers of cold agglutinins. There was no history of common risk factors of atherosclerosis, such as hypertension, diabetes mellitus, coronary artery disease or smoking. After being exposed to warm temperature, and with corticosteroid therapy and blood transfusion, the patient’s symptoms relieved rapidly.

**Conclusion:**

We report here the first case of cerebral infarction probably due to the cold agglutinin disease. The underlying mechanism of cold agglutinins in the pathogenesis of acute ischemic stroke needs to be investigated further.

## Background

Cold agglutinin disease, which was initially described in 1957 [1], is an autoimmune disease characterized by the presence of high concentrations of circulating antibodies, usually immunoglobulin M (IgM), directed against red blood cells, causing autoimmune hemolytic anemia, specifically at low body temperatures, typically 28–31 °C [2]. At body temperatures of 28–31 °C, antibodies bind to the polysaccharide region of glycoproteins present on the surface of red blood cells. Binding of antibodies to red blood cells activates the classical pathway of the complement system with resultant damage of red blood cells by the membrane attack complex, leading to intravascular hemolysis. To the best of our knowledge, there are no reports on the relationship between cold agglutinin disease and cerebral infarction. We describe here a patient with acute ischemic stroke in whom cold agglutinins were positive during the acute phase of the illness and the symptoms of stroke dramatically resolved upon exposure to warm temperature.

## Case presentation

On a freezing cold morning, a 71-yr-old male of Han nationality who was retired, but worked as a community volunteer, was brought to the neurology emergency with a history of sudden onset of left-sided weakness and difficulty in speaking while he was performing road patrol. Approximately an hour and a half had elapsed since the onset of symptoms before he was brought to the emergency room.

As for the past medical history, he had undergone a surgical procedure involving left kidney, left ureter and bladder for treatment of transitional cell carcinoma of bladder, approximately 8 months ago. One month after the surgical procedure, the results of laboratory investigations, such as complete blood count, kidney and liver function tests were within normal limits. There was no history of common risk factors of atherosclerosis, such as hypertension, diabetes mellitus, coronary artery disease and smoking.

He was conscious, had pallor, the blood pressure was 150/80 mmHg and the blood sugar level was 6.5 mmol/L. Chest and cardiac examination was unremarkable. The neurological examination demonstrated a left-sided hemiparesis with clumsy speech. The muscle strength of the left limb was grade III and the left Chaddock’s sign was positive. The patient’s upper and lower extremities were very cold, and the finger tip oxygen saturation was 76 %. However, the arterial blood gas analysis revealed the oxygen saturation of 94 % without supplementary oxygen.

A computed tomography (CT) scan of the brain revealed absence of hemorrhage, hence the diagnosis of acute ischemic stroke was highly probable. Routine blood test revealed a haemoglobin of 58 g/L, platelet count of 124 × 10^9^/L,white blood cell count of 7.55 × 10^9^/L, however, the number of red blood cells, hematocrit, mean corpuscular volume could not be measured due to the interference by agglutinated erythrocytes. The low level of hemoglobin combined with presence of pallor suggested severe anemia. On coagulation study, d-dimmer was found to be 19.17 mg/L and fibrin degradation product (FDP) was found to be 101.2 mg/L, indicating secondary fibrinolysis.

Due to the low level of hemoglobin, intravenous thrombolysis was contraindicated and the patient was hospitalized and treated with gastrodin. On the same day, the neurological symptoms of the patient dramatically recovered with the recovery of strength in the left limb (grade V), and the development of relatively clear speech. During this time, the patient was kept in a warm room where the temperature was maintained at 24 °C. However, the patient complained of severe back pain and passage of dark colored urine. Urine examination revealed the presence of hemoglobinuria, on the basis of which we suspected acute intravascular hemolysis.

**Fig. 1:**
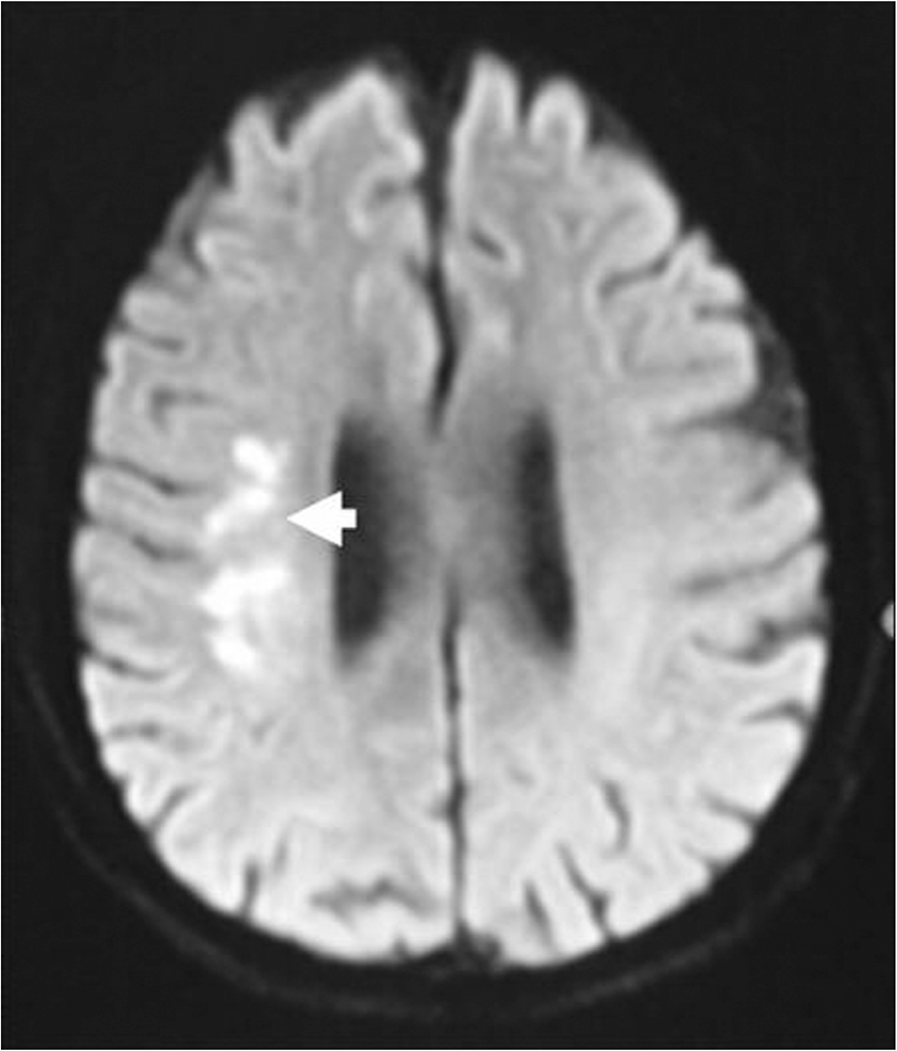
Diffusion weighted imaging of the magnetic resonance axial images showed high signal of the right basal ganglia and corona radiata indicating acute ischemic infarction (arrow)

The direct Coombs test was performed, which was negative for anti-globulin immunoglobulin G, but positive for anti-C3d, indicating the possibility of autoimmune hemolytic anemia. To rule out the possibility of connective tissue disease, tests for antinuclear antibody (ANA), antineutrophil cytoplasmic antibodies (ANCA), and antibodies to a spectrum of extractable nuclear antigens (ENA), such as smooth muscle (Sm), ribonucleoprotein (RNP), Sjogren's syndrome A (SSA), Sjogren's syndrome B (SSB), DNA topoisomerase I (Scl70), and histidyl-tRNA synthetase (Jo-1) were performed, which were found to be negative. To rule out multiple myeloma, serum and urine protein electrophoresis was performed, which were negative. The cold agglutinins were positive with a titer more than 1:1000. The abdominal enhanced computer tomography (CT) revealed no signs of relapse of bladder cancer. The cranial magnetic resonance imaging (MRI) showed the presence of acute infarction in the area of right basal ganglia and corona radiata (Fig. 1).

**Fig. 2:**
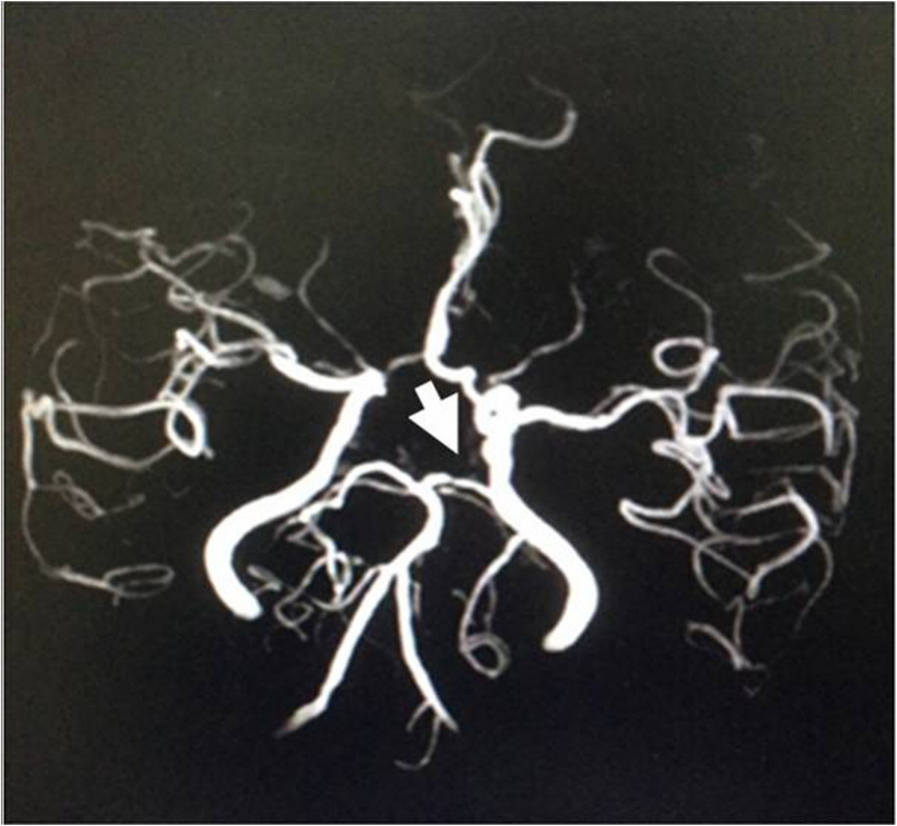
Cranial magnetic angiography demonstrating no dramatic stenosis of the intracranial large artery except mild to moderate stenosis of left posterior cerebral artery (arrow) which could not fully account for the ischemic lesion

On magnetic resonance angiography (MRA), we could not demonstrate severe stenosis of intracranial internal carotid artery; however, there was mild to moderate stenosis of the left posterior cerebral artery (Fig. 2). The extracranial internal carotid artery was found to be normal on carotid sonography.

Based on the above mentioned findings, a diagnosis of the cold agglutinin disease and ischemic stroke were made. The patient was transfused with 2 units of the washed red blood cells, and received corticosteroids, sodium bicarbonate and intravenous fluid therapy. After one day of corticosteroid therapy the back pain was dramatically relieved. The urine of the patient became progressively less dark. After one week of therapy, the titer of cold agglutinins decreased to < 1: 10 and the patient’s symptoms completely disappeared.

## Discussion

Based on the clinical features, findings of laboratory investigations and imaging studies, diagnosis of cold agglutinin disease with acute ischemic stroke was made. Cranial MRI demonstrated that the etiology of the ischemic stroke was most likely due to the hypoperfusion in the right middle cerebral artery (MCA) territory, with the large arteries being almost normal with no evidence of cardioembolism. In addition, common risk factors of atherosclerosis, such as hypertension, diabetes mellitus, coronary artery disease and smoking, were negative. High resolution MRI (HR-MRI) of the right MCA should have been performed to check whether there was lesion in the MCA vascular wall. However, due to technical limitations, HR-MRI could not be performed in this patient. However, even if there was lesion in the MCA vascular wall, it may have contributed synergistically with cold agglutinins in the pathogenesis of infarction. In other words, the cold agglutinins may have aggravated the MCA watershed infarction. Modi *et al.* [3] reported the role of cold agglutinins in the pathogenesis of Takayasu’s disease, in which there is large artery involvement. Otsuka *et al.* [4] reported that the cold agglutinins were responsible for necrotizing peripheral vasculitis leading to mononeuropathy. To the best of our knowledge, there is no report about the relationship between cold agglutinin disease and ischemic stroke. Cold induced circulatory symptoms are considered typical for cold agglutinin disease and more than 90 % of patients report symptoms of moderate acrocyanosis to severe Raynaud’s phenomena, precipitated even by very slight exposure to cold [2]. Since the temperature of the brain is relatively stable, it usually unaffected by the surrounding temperature. Why the cold agglutinins were activated in the central nervous system still remains a mystery to us, which requires further investigation.

Cold agglutinin disease has traditionally been classified into a primary or idiopathic and secondary, with primary type being unrelated to underlying conditions, and secondary type being associated with malignant disease, most often lymphoma [2]. Ulvestad *et al.* [5] reported occurrence of hemolysis after infection and trauma in a patient with cold agglutinin disease. Jeong *et al.* [6] reported a patient with urinary bladder carcinoma and cold agglutinin disease, which got precipitated intraoperatively due to exposure to low temperature. The cold agglutinin disease in our patient may have been associated with the history of urinary bladder carcinoma.

## Conclusion

This is the first report of acute cerebral infarction probably due to the cold agglutinins syndrome, even if the mechanisms are not entirely understood and an investigation into the role of cold agglutinins in the pathogenesis of acute ischemic stroke is necessary.

## Consent

Written informed consent was obtained from the patient for publication of this case report and any accompanying images. A copy of the written consent is available for review by the Editor-in-Chief of this journal.

## Abbreviations

ANCA: Antineutrophil cytoplasmic antibodies
CT: Computed tomography
ENA: Extractable nuclear antigens
FDP: Fibrin degradation product
HR-MRI: High resolution magnetic resonance imaging
MRA: Magnetic angiography
MRI: Magnetic resonance imaging
